# Identification of Anisomerous Motor Imagery EEG Signals Based on Complex Algorithms

**DOI:** 10.1155/2017/2727856

**Published:** 2017-08-09

**Authors:** Rensong Liu, Zhiwen Zhang, Feng Duan, Xin Zhou, Zixuan Meng

**Affiliations:** College of Computer and Control Engineering, Nankai University, Tianjin 300350, China

## Abstract

Motor imagery (MI) electroencephalograph (EEG) signals are widely applied in brain-computer interface (BCI). However, classified MI states are limited, and their classification accuracy rates are low because of the characteristics of nonlinearity and nonstationarity. This study proposes a novel MI pattern recognition system that is based on complex algorithms for classifying MI EEG signals. In electrooculogram (EOG) artifact preprocessing, band-pass filtering is performed to obtain the frequency band of MI-related signals, and then, canonical correlation analysis (CCA) combined with wavelet threshold denoising (WTD) is used for EOG artifact preprocessing. We propose a regularized common spatial pattern (R-CSP) algorithm for EEG feature extraction by incorporating the principle of generic learning. A new classifier combining the *K*-nearest neighbor (KNN) and support vector machine (SVM) approaches is used to classify four anisomerous states, namely, imaginary movements with the left hand, right foot, and right shoulder and the resting state. The highest classification accuracy rate is 92.5%, and the average classification accuracy rate is 87%. The proposed complex algorithm identification method can significantly improve the identification rate of the minority samples and the overall classification performance.

## 1. Introduction

Brain-computer interface (BCI) provides an efficient communication bridge between the human brain and external manageable devices [1]. Among the signal-controlling BCI sources, the P300 [2], steady-state visual-evoked potential (SSVEP) [3], and motor imagery (MI) [4] signals are the most commonly used. In contrast to SSVEP and P300, MI is a self-induced brain activity, which is initiated by imaging certain limbs or other body parts to move without the help of outside inducing factors [5]. An MI BCI system was first used based on this feature to assist humans with severe disabilities [6]. This system is also used for humanoid controls [7], entertainment game designs [8], and aircraft flight controls [9]. However, the performance of this system is largely dependent on the number of MI motion commands that can be precisely classified.

The cerebral cortex of left-handers and right-handers is anisomerous [10]. Therefore, cerebral cortex activities often present evident differences and cannot be easily distinguished when right-handers imagine symmetric limb movements [11, 12]. Our study aims to analyze and recognize four anisomerous MI states, namely, imaginary movements with the left hand, right foot, and right shoulder and the resting state.

In general, MI pattern recognition systems involve raw MI EEG signal preprocessing, feature extraction, and pattern classification. However, subjects experience difficulty in avoiding eye movements and consequently produce electrooculogram (EOG) artifacts in raw MI EEG signals [13]. The obtained raw MI EEG signals are mainly affected by the vertical EOG (VEOG) signals generated by blinking eyes. The preprocessing algorithms for EEG signals mainly include time domain filtering, blind source separation [14], and time-frequency domain analysis methods. Time domain methods, such as the low-pass filter method and band-pass filter method [15], have been used to eliminate EOG artifacts [16, 17]. However, time domain filtering methods cannot effectively remove the majority of the EOG artifacts. Vergult et al. [18] used blind source separation and canonical correlation analysis (CCA) to effectively denoise EOG artifacts from raw MI EEG signals, but the CCA algorithm should be able to artificially recognize the artifact components. Hsu et al. [19] used a time-frequency domain analysis method called discrete wavelet transform (DWT) to denoise EOG artifacts from raw EEG signals. The multiresolving feature of DWT enables nonstationary EEG signals to be considered. However, a small portion of the EOG artifacts remains in the EEG signals after the DWT denoise preprocessing is completed. Thus, a more effective preprocessing algorithm should be developed to denoise EOG artifacts.

Feature extraction is another critical step in MI pattern recognition. Common EEG features include those in the time domain, frequency domain, time-frequency domain, and spatial domain [20]. Time domain analysis is mainly conducted to extract EEG features because MI EEG signals are recorded in the time domain. For example, Khushaba et al. [21] extracted EEG features from the time domain to form a set of features that was relevant to the limb position. EEG signals also contain various frequency components. Prasad et al. [22] used power spectral density as an EEG feature. Time-frequency domain analysis methods can integrate the advantages of time domain and frequency domain analysis methods. Wang et al. [23] applied a wavelet packet transform method to extract the time and frequency information in EEG signals. However, univariate and integrated analysis methods using the time domain and frequency domain are not appropriate for multichannel EEG feature extraction [24].

After preprocessing raw MI EEG signals and extracting the features, we aimed to develop an appropriate classifier to precisely categorize the MI motion commands. Common classification algorithms for EEG features include the linear distance discriminant [25], support vector machine (SVM) [26], clustering algorithms [27], Bayesian classifiers, and back propagation neural network (BPNN) classifiers [28]. However, the classifiers exhibit poor performance when the EEG features overlap with one another.

Considering previous studies, we propose a novel MI pattern recognition system for classifying MI EEG signals. We use the Butterworth band-pass filter to extract EEG signals having frequencies of 8–30 Hz during the preprocessing of raw EEG signals. We then apply a CCA algorithm that integrates a wavelet threshold denoising (WTD) algorithm to form a compound algorithm called the wCCA algorithm and to process the extracted frequency band signals. We also use a regularized common spatial pattern (R-CSP) algorithm by incorporating the principle of generic learning [29] to extract the EEG features in the spatial domain. This approach can effectively extract connotative spatial information from multichannel EEG signals and reduce the data dimension based on minority samples. We combine the *K*-nearest neighbor (KNN) and SVM methods, which we call KNN-SVM, to classify the EEG features. We compare the KNN-SVM classifier to several classifiers to validate its classification performance.

The remainder of this paper is organized as follows.  Section 2 describes the EEG signal acquisition.  Section 3 introduces the raw EEG signal preprocessing.  Section 4 explains feature extraction with the R-CSP algorithm.  Section 5 discusses the KNN-SVM classifier and compares it with several classifiers.  Section 6 presents our experimental results and a discussion.  Section 7 provides the conclusions and recommends concepts for future studies.

## 2. EEG Signal Acquisition

**Figure 1:**
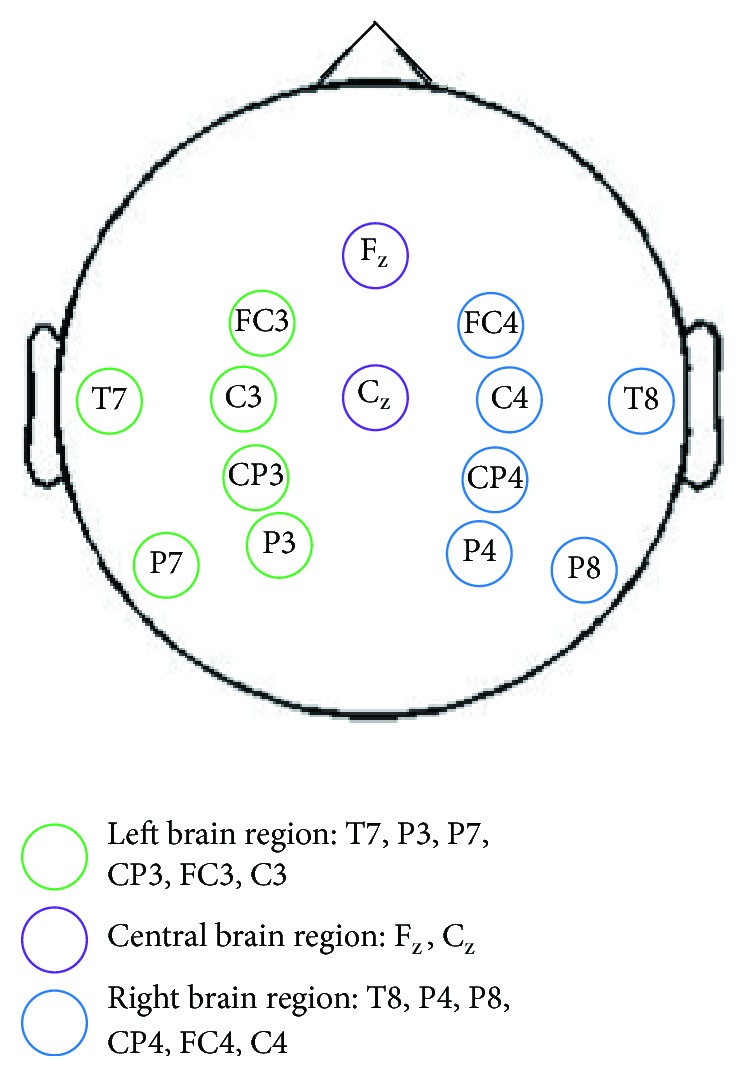
The positions of the EEG electrodes.

We selected 14 Ag/AgCl electrodes that were relevant to the MI brain region based on the Brodmann brain function partition and international 10/20 electrode lead system [30, 31]. Among the 14 electrodes, two (F_Z_, C_Z_) were placed in the central brain region, six (T7, P3, P7, CP3, FC3, and C3) were in the left brain region, and six (T8, P4, P8, CP4, FC4, and C4) were located in the right brain region. The electrodes in the left and right brain regions are symmetric (Figure 1). Bipolar lead modes with two electrodes were used to record vertical EOG (VEOG) signals: one electrode was placed above the left eyebrow, and the other electrode was placed on the lower edge of the left eye socket. Monopolar derivations were used throughout the recordings. In this process, the left mastoid and forehead served as the reference and ground, respectively. The signals were sampled at 256 Hz, and an additional 50 Hz notch filter was enabled to suppress the power line interference by using a g.tec device (g.tec medical engineering GmbH, Schiedlberg, Austria).

**Figure 2:**
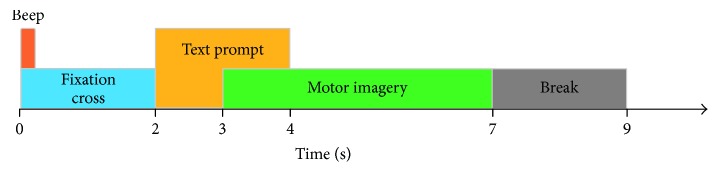
Timing scheme of the EEG signal recording.

A subject sat on a relaxing chair, and the subject's arms were placed in a relaxed position on his legs. The paradigm consisted of four different tasks, namely, imaginary movements with the left hand (LH), right foot (RF), right shoulder (RS), and the resting state (R). At the beginning of a trial (*t* = 0 s), a fixation cross “+” was displayed on a black screen. In addition, a short acoustic warning tone was presented. After two seconds (*t* = 2 s), a text prompt for the left hand (LH), right foot (RF), right shoulder (RS), or resting state (R) was displayed in the center of the screen and remained on the screen for 2 s. This prompted the subject to perform the desired MI task. The subject was asked to continue performing the MI task until the fixation cross “+” disappeared from the screen at *t* = 7 s. A short break followed, with a blank screen lasting for two seconds. The paradigm is illustrated in Figure 2.

Five healthy subjects, namely, three men (Subjects A, B, and D) who were 30, 25, and 23 years of age, respectively, and two women (Subjects C and E) who were 21 and 23 years of age, respectively, participated in the experiment and performed the four MI tasks. Subject A was left-handed, and the other subjects were right-handed. Each MI motion state was recorded for one session, and altogether four sessions were recorded for each subject. Each session consisted of 60 trials separated by short breaks (lasting a couple of minutes) breaks. For each state, 50 trials were selected for training and the remaining 10 trials were used for testing. In total, 240 trials were performed per subject.

## 3. Raw EEG Signal Preprocessing

**Figure 3:**
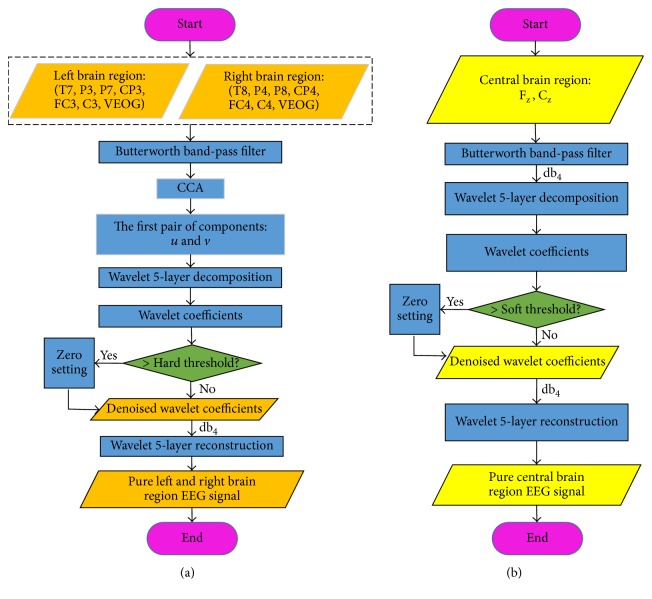
Structure of the EEG signal preprocessing. (a) Denoising of the twelve symmetrical electrodes using wCCA. (b) Denoising of the two central brain region electrodes using WTD.

For raw EEG signals, the Butterworth band-pass filter was used to extract the 8–30 Hz frequencies of the signals. The brain is a good conductor of electricity. As such, EOG signals spread from the forehead to the back of the head and thus traverse the entire head. We considered the different spatial distribution characteristics of the EEG and EOG signals. For twelve symmetrical electrodes (T7, T8, P3, P4, P7, P8, CP3, CP4, FC3, FC4, C3, and C4), we used the wCCA algorithm to examine the mixed signals in a new form. **X** represents the EEG signals collected from six electrodes in the left brain region, and **Y** denotes the six other electrodes in the right brain region. The VEOG signal was added to **X** and **Y**. The first pair of components was calculated through CCA decomposition and exhibited the highest correlation. The components can be regarded as the most communal ingredient between the left and right brain regions, which are composed of the EOG artifacts and a small number of high-frequency EEG components. Then, wavelet threshold denoising was performed to remove the EOG artifacts and maintain a small amount of high-frequency EEG components. Finally, pure EEG signals of twelve symmetric electrodes were obtained through wCCA algorithm processing. For two central brain region electrodes (F_Z_, C_Z_), the wavelet basis “db4” was used to conduct five-layer wavelet decomposition for the EEG signals of F_Z_ and C_Z_. Then, the wavelet soft threshold denoising function “wdencmp” was used to process the decomposed signal components. Next, the denoised signal components were used to reconstruct the pure EEG signals with the wavelet basis “db4.” The structure of the EEG signal preprocessing is shown in Figure 3.

### 3.1. wCCA Algorithm

Next, the derivation process of the wCCA algorithm was described in detail. Suppose that **X** = [**x**_1_^*T*^, …,**x**_6_^*T*^, **z**^*T*^]^*T*^ and **Y** = [**y**_1_^*T*^, …,**y**_6_^*T*^, **z**^*T*^]^*T*^ represent EEG signals that were collected from the left brain region and right brain region, respectively. Among them, **x**_1_^*T*^, …, **x**_6_^*T*^ and **y**_1_^*T*^, …, **y**_6_^*T*^ are two sets of 12 raw symmetric electrode EEG signals and **z**^*T*^ is the VEOG signal. After the centralization of **X** and **Y**, the new variables $\hat{X}$ and $\hat{Y}$ are obtained, respectively. We then find a linear combination of the points through CCA to obtain the new variables **U** and **V**, which exhibit the highest correlation.

(1) ui=ai,1x^1+ai,2x^2+⋯+ai,6x^6+ai,7z^=aiTX^,vi=bi,1y^1+bi,2y^2+⋯+bi,6y^6+bi,7z^=biTY^.

The obtained canonical correlation variables are the estimations of seven raw independent signals **u**_*i*_ and **v**_*i*_, *i* = 1,…, 7, respectively. The vectors **a**_*i*_ and **b**_*i*_, *i* = 1,…, 7 are obtained by calculating the maximum simple correlation coefficient. $C_{xx}=E\left[\hat{X}{\hat{X}}^{T}\right]$ and $C_{yy}=E\left[\hat{Y}{\hat{Y}}^{T}\right]$ are the autocovariance matrices. $C_{xy}=E\left[\hat{X}{\hat{Y}}^{T}\right]$ and $C_{yy}=E\left[\hat{Y}{\hat{X}}^{T}\right]$ are the cross-variance matrices.

(2) maxai,bi⁡ ρui,vimaxai,bi⁡covui,vivaruivarvi=maxai,bi⁡aiTCxybiaiTCxxaibiTCyybi,i=1,…,7,

where the constraints are

(3) aiTCxxai=1,biTCyybi=1.

The Lagrangian function is constructed to calculate the values of **a**_*i*_ and **b**_*i*_ under the premise that *ρ*(**u**_*i*_, **v**_*i*_) achieves the maximum value:

(4) Lai,bi=aiTCxybi−λ12aiTCxxai−1−λ22biTCyybi−1.

According to (1), **U** and **V** have the following forms:

(5) U=ATX^,V=BTY^.

The obtained canonical correlation variables **U** and **V** included seven independent components, **U** = [**u**_1_^*T*^, …,**u**_7_^*T*^]^*T*^ and **V** = [**v**_1_^*T*^, …,**v**_7_^*T*^]^*T*^. The vectors **a**_*i*_ and **b**_*i*_, *i* = 1,…, 7 are the *i*th columns of matrices **A** and **B**, respectively.

Next, **A**, **B**, *λ*_1_, and *λ*_2_ can be calculated based on (2) to (4). The first independent components **U** and **V** are called **u**_1_ and **v**_1_. Each component is composed of EOG artifacts and several valuable EEG signals. Then, we used a wavelet hard threshold noise reduction method. We first used the wavelet basis “db4” for five-layer signal wavelet decomposition for **u**_1_ and **v**_1_. Thus, we obtained five wavelet coefficients and one scale coefficient. Then, we set any coefficient that was higher that the threshold to zero, whereas we retained the value of any coefficient that was lower that the threshold. In 1994, Donoho proposed the VisuShrink method (the unified threshold denoising method). The threshold *k*_*j*_ for each coefficient is defined as follows:

(6) $$\begin{array}{c} k_{j}=\sqrt{2\ln\left(N_{j}\right)}d_{j},\;j=1,\ldots ,5, \end{array}$$

where *k*_*j*_ is the threshold for the *j*th coefficient and *N*_*j*_ is the number of elements for the *j*th coefficient. Donoho and Johnstone [32] proposed an estimation formula for the noise standard deviation in the wavelet domain ${\hat{\delta }}_{n}=\text{MAD}/0.6745$, where MAD is the median value of the subband wavelet coefficient. Thus, the standard deviation of the noise in wavelet domain *d*_*j*_ is defined as follows:

(7) $$\begin{array}{c} d_{j}=\frac{median\left(D_{j}\right)}{0.6745}, \end{array}$$

where *D*_*j*_ is the *j*th coefficient.

After the wavelet hard threshold noise reduction processing, the six processed coefficients were used for wavelet transform reconstruction with the wavelet basis “db_4_,” and we obtained the denoised independent component signals **u**_1new_ and **v**_1new_ which are the wavelet threshold denoised signal of **u**_1_ and **v**_1_, respectively. These signals and the other six independent components comprise **U**_new_ and **V**_new_.

According to (5), after calculating **U**_new_ and **V**_new_, we reconstructed the new variables ${\hat{X}}_{\text{new}}$ and ${\hat{Y}}_{\text{new}}$, which are the estimates of the pure EEG signals from the left brain region and right brain region, respectively.

(8) X^new=AT−1Unew,Y^new=BT−1Vnew.

Twelve pure electrodes with symmetric EEG signals were obtained. ${\hat{X}}_{\text{new}}$ represents the six EEG electrode signals from the left brain region, and ${\hat{Y}}_{\text{new}}$ represents the six EEG channel electrodes signals from the right brain region.

### 3.2. EEG Signal Denoising

**Figure 4:**
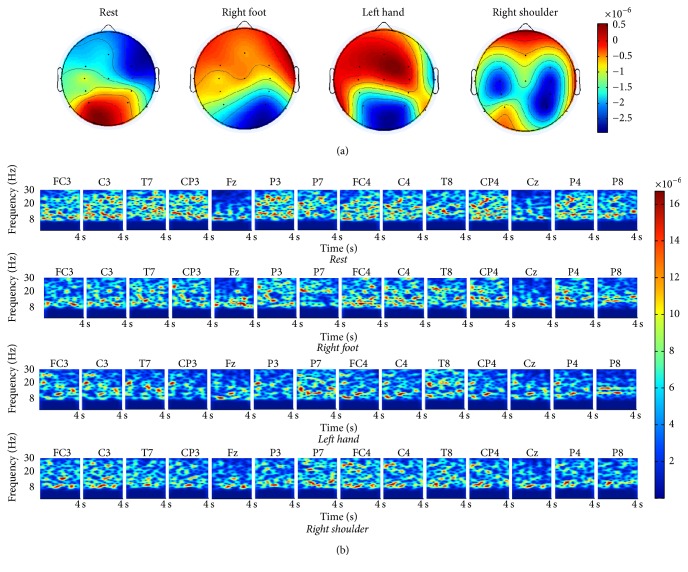
Brain topographic map and 14-channel spectrum map from Subject A. (a) Brain topographic map. (b) 14-channel spectrum map.

We constructed brain topographic maps of the four MI states to examine the topology of significant EEG features.  Figure 4(a) shows the brain topographic map of Subject A. The red color and blue color both indicated higher values within the corresponding state. Evident differences were observed among the four brain topography maps. Therefore, we can obtain good classification effectiveness. Furthermore, we constructed time-frequency maps to concretely obtain the activity degree of the 14 electrodes in each MI state.  Figure 4(b) shows the 8–30 Hz frequency spectrum chart for the 14 electrode EEG signals of each MI state from Subject A. Taking the resting state as an example, electrode F_Z_ exhibits the lowest activity degree, whereas electrode C3 exhibits the highest activity degree.

**Figure 5:**
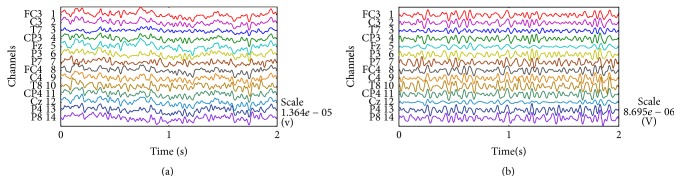
Raw and denoised signals. (a) Raw signals. (b) Denoised signals.

The 14-channel raw EEG signals are mixed with EOG signals; in particular, electrodes close to the eyes are particularly influenced by the EOG signals.  Figure 5(a) shows the time domain graph of the resting state from Subject A. In Figure 5(a), the EEG electrode signals of FC3, F_Z_, and FC4 fluctuate significantly, whereas the other electrode signals are less affected by the EOG signals because these electrodes are far from the eyes. We obtained the pure EEG signals after the EEG signal preprocessing is completed (Figure 5(b)). The denoised 14-channel EEG signals fluctuate only slightly.

## 4. Feature Extraction Using R-CSP

**Figure 6:**
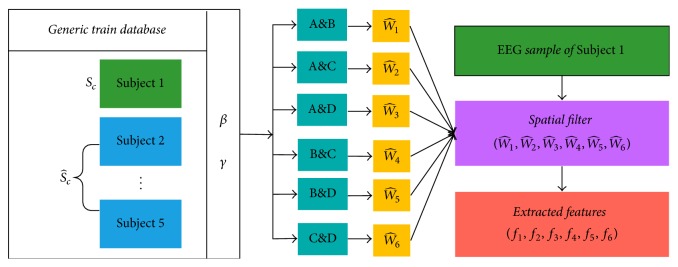
Structure of the feature extraction using R-CSP.

After the preprocessing of the raw EEG signals, we must extract the EEG features (Figure 6). The common space model (CSP) is more effective than the traditional time-frequency domain feature extraction method for extracting the differences in the spatial features of the two types of signals. However, the CSP algorithm is based on a large number of signal samples based on covariance estimation. Therefore, the feature extraction is affected by the number of samples available for training. In recent years, regularized discriminant analysis (RDA) has been used to solve small sample problems for linear and secondary discriminant analyses. The small-training-sample approach leads to biased estimates of the eigenvalues, and such problems can lead to instability in the feature extraction. Thus, two regularization parameters are used to address these undesirable features.

In this paper, we adopt the improved regularized common spatial pattern (R-CSP) algorithm by incorporating the principle of generic learning to extract the EEG features in the spatial domain. In R-CSP, we used the principle of generic learning to address one training sample problem. The training set of R-CSP uses a generic database that contains subjects that are different from those to be identified. The classifier is trained to extract the discriminant information from the subjects other than those that will be called on to perform recognition when in operation. The principle behind generic learning is that the discriminant information pertinent to the specific subjects (those to be identified) can be learned from other subjects because the EEG signals exhibit similar intrasubject variations. The R-CSP algorithm is an improved CSP algorithm. It can provide a good approach to overcoming outlier (such as noise) sensitivity and poor robustness, which are shortcomings of having a small number of samples. There are two parameters in the R-CSP algorithm, *β* and *γ*. The first regularization parameter controls the shrinkage of a subject-specific covariance matrix toward a “generic” covariance matrix to improve the estimation stability based on the principle of generic learning. The second regularization parameter controls the shrinkage of the sample-based covariance matrix estimation toward a scaled identity matrix to account for the bias due to the limited number of samples.

### 4.1. R-CSP Algorithm

We assume that there are *L* subjects who participated in the experiment. Assume that **G**_1_ and **G**_2_ are two kinds of MI tasks in the space multimodal evoked response signal matrix from the multichannel MI EEG signals. Their dimensions are *N* × *T*, where *N* is the number of EEG channels and *T* is the number of samples collected for each channel. **E** is a trial of *N* × *T* dimensions MI EEG signals from MI task **G**_1_ or **G**_2_.

The normalized sample covariance matrix **S** of a trial **E** is obtained as follows:

(9) $$\begin{array}{c} S=\frac{EE^{T}}{\text{trace}\left(EE^{T}\right)}. \end{array}$$

The two MI tasks of EEG signals are indexed by *c* = {1,2}. For simplicity, we assume that there are *M* trials in each class that are available for training for a subject of interest, which are indexed by *m* as in **E**_(*c*, *m*)_, where *m* = 1,…, *M*. Thus, each trial has a corresponding covariance matrix **S**(*c*, *m*).

The average spatial covariance matrix for each class is then calculated as follows:

(10) $$\begin{array}{c} {\overset{-}{S}}_{c}=\frac{1}{M}\overset{M}{\underset{m=1}{\sum }}S\left(\;c,m\;\right),\;c=\left\{1,2\right\}. \end{array}$$

Next, the regularization technique is introduced into the equation. The regularized average spatial covariance matrix for each class is calculated as

(11) $$\begin{array}{c} {\hat{\Sigma }}_{c}\left(\;\beta ,\gamma \;\right)=\left(\;1-\gamma \;\right){\hat{\Sigma }}_{c}\left(\;\beta \;\right)+\frac{\gamma }{N}tr\left[\;{\hat{\Sigma }}_{c}\left(\;\beta \;\right)\;\right]\cdot I, \end{array}$$

where *β*  (0 ≤ *β* ≤ 1) and *γ*  (0 ≤ *γ* ≤ 1) are two regularization parameters, I is an identity matrix of size *N* × *T*, and ${\hat{\Sigma }}_{c}(\beta )$ is defined as follows:

(12) $$\begin{array}{c} {\hat{\Sigma }}_{c}\left(\;\beta \;\right)=\frac{\left(1-\beta \right)\cdot S_{c}+\beta \cdot {\hat{S}}_{c}}{\left(1-\beta \right)\cdot M+\beta \cdot \hat{M}}. \end{array}$$

In (12), **S**_*c*_ is the sum of the sample covariance matrices for all *M* training trials in class *c*  (*c* = 1,2), and ${\hat{S}}_{c}$ is the sum of the sample covariance matrices for a set of $\hat{M}\left\{M\times \left(L-1\right)\right\}$ generic training trials from the other (*L* − 1) subjects in class *c*.

(13) Sc=∑m=1MSc,m,S^c=∑m^=1M^Sc,m^.

Next, the composite spatial covariance is formed and factorized as

(14) $$\begin{array}{c} {\hat{\Sigma }}_{c}\left(\;\beta ,\gamma \;\right)={\hat{\Sigma }}_{1}\left(\;\beta ,\gamma \;\right)+{\hat{\Sigma }}_{2}\left(\;\beta ,\gamma \;\right)=\hat{\text{U}}\hat{\text{Λ}}{\hat{\text{U}}}^{T}, \end{array}$$

where $\hat{\text{U}}$ is the matrix of eigenvectors and $\hat{\text{Λ}}$ is the diagonal matrix of corresponding eigenvalues. In this paper, we adopt the convention that the eigenvalues are sorted in descending order.

Next, the whitening transformation is obtained as follows:

(15) $$\begin{array}{c} \hat{\text{P}}={\hat{\text{Λ}}}^{-1/2}{\hat{\text{U}}}^{T}. \end{array}$$

${\hat{\Sigma }}_{1}\left(\beta ,\gamma \right)$ and ${\hat{\Sigma }}_{2}\left(\beta ,\gamma \right)$ are whitened as follows:

(16) Σ~1β,γ=P^Σ^1β,γP^TΣ~2β,γ=P^Σ^2β,γP^T.

${\tilde{\Sigma }}_{1}\left(\beta ,\gamma \right)$ can then be factorized as follows:

(17) $$\begin{array}{c} {\tilde{\Sigma }}_{1}\left(\;\beta ,\gamma \;\right)=\hat{\text{B}}{\hat{\Lambda }}_{1}{\hat{\text{B}}}^{T}. \end{array}$$

The full projection matrix of ${\tilde{\Sigma }}_{1}\left(\beta ,\gamma \right)$ is formed as follows:

(18) $$\begin{array}{c} {\hat{\text{W}}}_{0}={\hat{\text{B}}}^{T}\hat{\text{P}}. \end{array}$$

For the most discriminative patterns, only the first and last *α* (we set *α* = 2) columns of ${\hat{\text{W}}}_{0}$ are retained to form $\hat{W}$, which is of size *N* × *Q*, where *Q* = 2*α*. For the feature extraction, a trial **E** is first projected as follows:

(19) $$\begin{array}{c} \hat{\text{Z}}={\hat{W}}^{T}E. \end{array}$$

Then, a *Q*-dimensional feature vector $\hat{y}$ is formed from the variance of the rows of $\hat{\text{Z}}$ as follows:

(20) $$\begin{array}{c} {\hat{y}}_{q}=\log\left(\;\frac{var\left({\hat{z}}_{q}\right)}{\sum_{q=1}^{Q}var\left({\hat{z}}_{q}\right)}\;\right), \end{array}$$

where ${\hat{y}}_{q}$ is the *q*th component of $\hat{y}$, ${\hat{z}}_{q}$ is the *q*th row of $\hat{\text{Z}}$, and $var({\hat{z}}_{q})$ is the variance of vector ${\hat{z}}_{q}$.

However, in this study, we analyzed four MI states, assuming that the four states are A, B, C, and D. We converted the four-classification tasks (A&B&C&D) into six two-classification-tasks: (A&B), (A&C), (A&D), (B&C), (B&D), and (C&D). Thus, six spatial filters are generated: ${\hat{\text{W}}}_{1}$, ${\hat{\text{W}}}_{2}$, ${\hat{\text{W}}}_{3}$, ${\hat{\text{W}}}_{4}$, ${\hat{\text{W}}}_{5}$, and ${\hat{\text{W}}}_{6}$. Finally, the four state signals are sequentially passed through the six spatial filters, and the feature vectors are obtained.

### 4.2. Feature Selection

**Figure 7:**
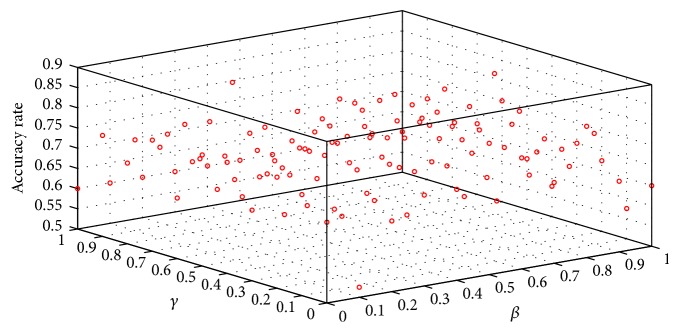
Classification results with different combinations of *β* and *γ* using R-CSP from Subject A.

We constructed a six-spatial-filter group using the R-CSP algorithm. After the EEG signals of four MI states were extracted by the six-spatial-filter group, the diversity-maximized feature vectors of 24 (6 × *Q*) were obtained. To optimize the performance of the R-CSP algorithm, we explored the effect of classification with different combinations of *β* and *γ*. R-CSP with *β* = *γ* = 0 is equivalent to the classical CSP. We calculated 121 classification results by the outer product of *β* = [0 : 0.1 : 1] and *γ* = [0 : 0.1 : 1].  Figure 7 shows the 121 classification results with different combinations of *β* and *γ* from Subject A. Then, we determined *β* and *γ* values that corresponded to the maximum classification accuracy using the KNN-SVM algorithm. Five subjects participated in the experiment and performed the four MI motions. There were 240 trials for each subject, that is, 200 trials for training and 40 trials for testing. Incorporating the principle of generic learning, for each subject, the training set is 1,000 training trials, which is composed of the subject's own 200 training trials and 800 training trials from the other four subjects.

To verify the classification effectiveness of the feature extraction, we first used CSP and R-CSP to extract the features separately. Then, we used KNN-SVM to classify the extracted features.  Table 1 shows the different classification results using CSP and R-CSP. The classification accuracy rates (AC) of subjects A, B, and D, separately, were improved by 5, 7.5, and 10 percentage points, respectively. The classification accuracy rate of Subject E remained at the same level as CSP. The classification accuracy rate of Subject C is reduced by 5 percentage points. Overall, the feature extraction performance of R-CSP is better than that of CSP.

## 5. Classification Using KNN-SVM

The sample feature points of the four MI states indicate that the tested EEG signals can cross or overlap. The KNN method is a mature classification algorithm. The concept of this method is that, for a sample of interest, if the *K* most similar samples in the feature space belong to a particular category, then the sample of interest also belongs to this category. Because the KNN method mainly depends on the samples that are adjacent, it is limited compared with the method of discriminant domain for determining the category. Thus, the KNN method is more suitable than the other methods for crossed or overlapping samples. However, the KNN method classifier uses local information for prediction. Thus, KNN lacks good generalization ability under small sample conditions, and the classification results are easily affected by noise.

The SVM is a machine learning algorithm that is based on statistical learning theory. Specifically, the SVM is based on the principle of structural risk minimization, which effectively avoids the problems that exist in traditional learning methods, such as learning, dimension disaster, and local minima, and it still has good generalization ability under the condition of having a small sample size. In particular, the SVM is superior to other classification methods in solving two types of classification problems. However, the classification effect is not suitable for crossed or overlapping samples. The use of the SVM for multiclass classification remains limited.

**Figure 8:**
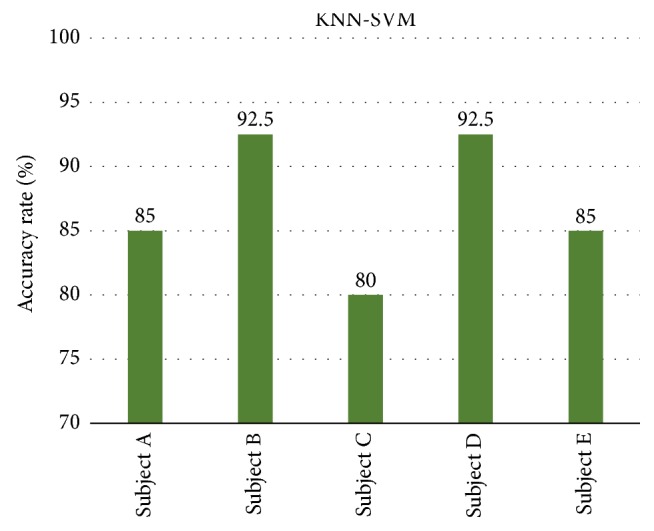
Accuracy results for the KNN-SVM from five subjects.

**Figure 9:**
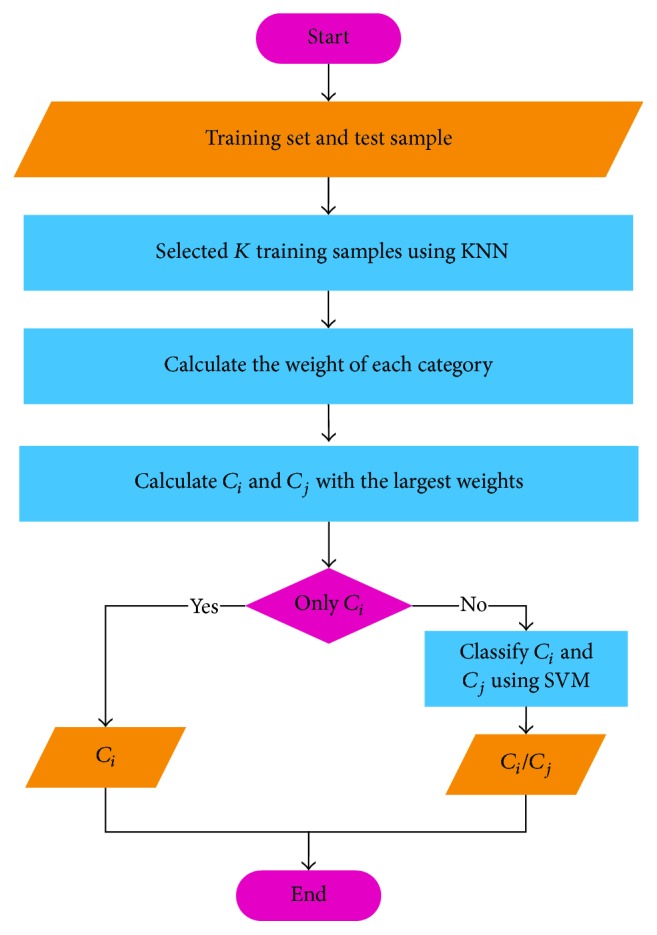
Structure of the KNN-SVM algorithm for feature classification.

Therefore, the KNN algorithm is used to establish the classification framework based on the KNN algorithm such that the KNN algorithm outputs the two most likely classification categories as the rough classification result, which is then input into the SVM for the second classification to obtain the final classification result. This new composite algorithm is called the KNN-SVM method. The new composite algorithm KNN-SVM can handle crossed or overlapping sample sets and still maintain good generalization ability under small sample conditions.  Figure 8 shows the accuracy results of KNN-SVM from five subjects. The classification accuracy rate varies from 80% to 92.5%. Subjects B and D have the best classification effects, with accuracies of up to 92.5%. The KNN-SVM algorithm shows good classification effectiveness. The steps of the KNN-SVM algorithm are as follows (see Figure 9).

**Step 1 .**

Calculate the cosine angle distance between the sample and the training set for each sample and obtain the first *K* training samples.

**Step 2 .**

Calculate the weight of each category of the selected *K* training samples.

**Step 3 .**

The two classes of *C*_*i*_ and *C*_*j*_ with the largest weights are selected as a result of the rough classification. If the classification result of the KNN algorithm is only one *C*_*i*_, then the instance is directly classified as *C*_*i*_; otherwise, *C*_*i*_ and *C*_*j*_ are two types of results using the one-against-one SVM for the final classification results of the two classifications.

## 6. Experimental Results and Discussion

In this study, the following three steps are involved in the MI pattern recognition system: (1) preprocessing of raw EEG signals; (2) extraction of the features of each state of the EEG signal; and (3) building a pattern recognition classifier. Five healthy subjects participated in the experiments. Each subject had to execute the four proposed MI states in the same experimental environment. The 240 trials of EEG signals obtained from each subject were divided into two sets, namely, the training trials and testing trials.

**Figure 10:**
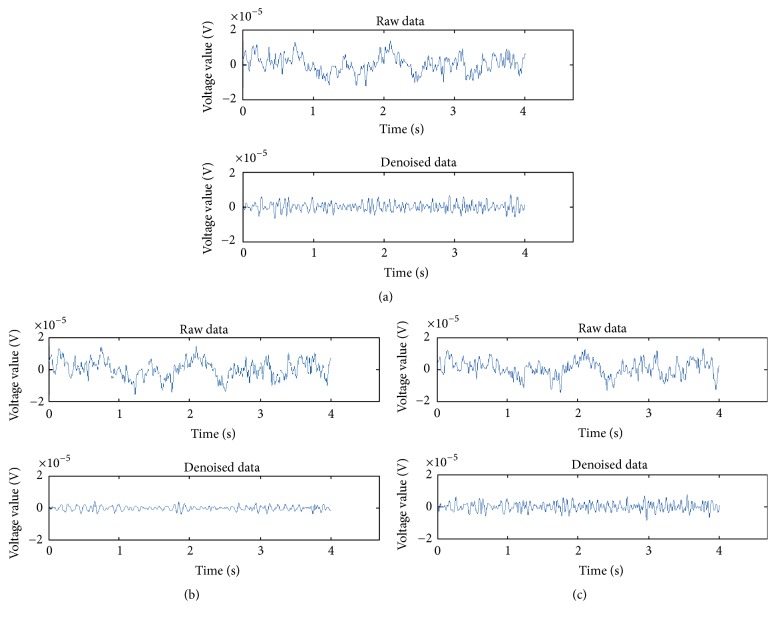
Raw and denoised signals of the three electrodes closest to the eyes. (a) FC3. (b) Fz. (c) FC4.

During the preprocessing of the raw EEG signals, we first used a notch filter to suppress the 50 Hz power frequency interference and the Butterworth band-pass filter to extract the 8–30 Hz frequencies of the EEG signals. Then, we used the wCCA and WTD algorithm to separate the EOG artifacts from the raw EEG signals (Figure 3). To demonstrate the effects of the EEG signal preprocessing, we consider the three electrode signals closest to the eyes before and after the preprocessing in Figure 10. The three raw electrode signals are mixed EOG signals and fluctuate significantly. After the preprocessing, the obtained three pure electrode signals fluctuate only slightly.

**Figure 11:**
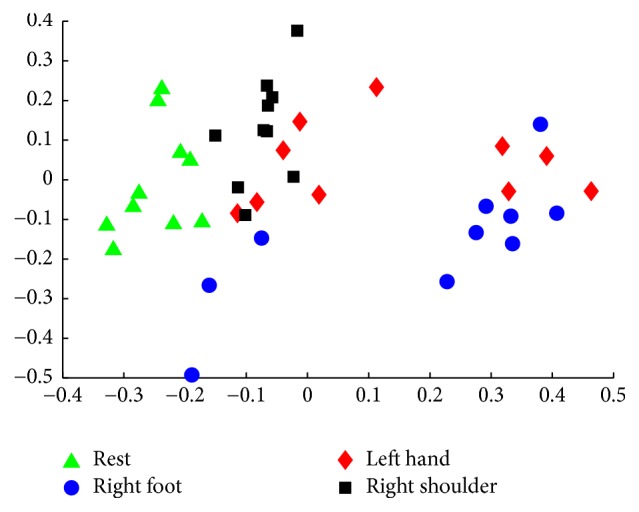
Sample feature points of four states using R-CSP from Subject A.

For the EEG feature extraction process, we used R-CSP to extract the EEG features in the spatial domain.  Figure 11 shows the scattering of the feature points for the 40 test samples of Subject A. The sample feature points of the LH are crossed or overlapping with feature points of the RS and RF. The classification accuracies of the five subjects for the four MI states are maximum with the best combination of *β* and *γ*, as shown in Table 1. Compared with the CSP, the classification accuracy of three subjects (A, B, and D) is higher using R-CSP, and only one subject (C) exhibited a slight decrease in the classification accuracy.

After training the classifier with the training set data, we used the test data as the input of the KNN-SVM to verify the classification accuracy rate.  Figure 8 shows the accuracy results of KNN-SVM for the five subjects. In addition, the classification results of KNN-SVM for the four MI states are provided in Table 2. For the five subjects, the accuracy rates of the resting (R) state and right foot (RF) state vary from 80% to 100%, and thus, KNN-SVM produces the best average classification accuracy of 92%. The accuracy rate of the right shoulder (RS) from Subject C has a low accuracy (70%), and thus, the accuracy rate of the RS has the second highest accuracy rate. For the left hand (LH), three subjects (A, C, and D) have low accuracy, and therefore, the average accuracy rate of the LH is the lowest (74%).

**Figure 12:**
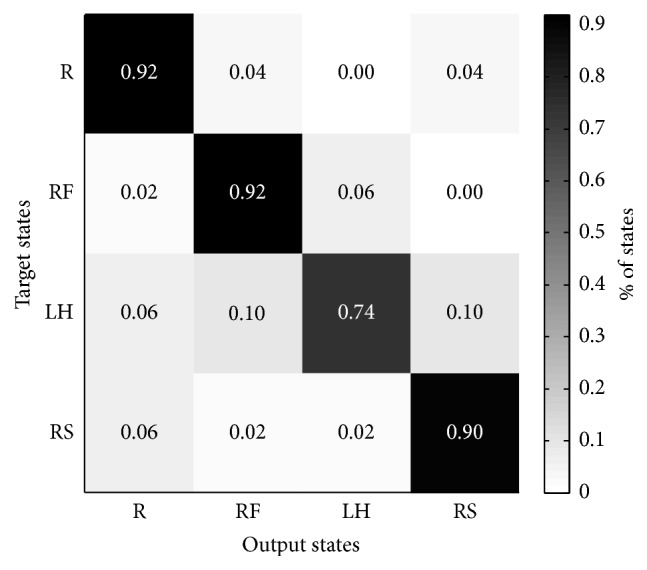
Confusion matrix for the recognition of MI states by KNN-SVM.

The confusion matrix is used to verify the actual discrimination success of the proposed method. If an MI state is often misconstrued as another state, then special pattern recognition efforts should be applied to address the complex problems related to the MI states.  Figure 12 shows the confusion matrix for the MI state categorizations by KNN-SVM. Using this matrix, the discrimination among the various MI states of all of the subjects can be evaluated in depth. Three MI states (R, RF, and RS) have good classification effectiveness, and only the LH has a low accuracy rate (74%). The misidentification of the LH state is mainly concentrated on the RF and RS. The confusion matrix illustrates that the KNN-SVM classifier is highly precise.

After EEG feature extraction using R-CSP, a suitable pattern recognition classifier is required. To confirm the good classification performance of the KNN-SVM classifier, we compare it with five commonly used classifiers (Table 3). We used those classifiers to classify the four MI states using the same sample data. Table 3 shows the classification results of six different classifiers. The KNN-SVM classifier has the highest average classification accuracy rate (87%), and the naive Bayes classifier has the lowest accuracy rate (69.5%). Among the six classifiers, the accuracy rates of KNN-SVM from the five subjects are all above 80%. In contrast, the accuracy rate of the Naive Bayes classifier is above 80% for only one subject (D). In addition, the standard deviation of KNN-SVM is the smallest, and thus, the KNN-SVM classifier is highly reliable. The performance of KNN-SVM is also significantly more efficient than those of the other five commonly used classifiers.

In this study, we adopted 16 EEG sensors to classify four MI states. Furthermore, we compared the results with those in previous studies to verify the contribution of our proposed EEG pattern recognition system [17, 32–34], as shown in Table 4. Additional EEG sensors can contribute to the quantity and quality of the MI state classification. However, increasing the number of sensors increases the complexity of the classification algorithms and deteriorates the stability performance of the EEG pattern recognition system. Furthermore, more sensors cause discomfort for the subjects. In Table 4, a minimum of 22 electrodes is required to recognize four MI states, but we utilized 16 sensors. In addition, the proposed method provides more effective classifications than the other methods.

## 7. Conclusions

We proposed a novel MI pattern recognition system for classifying four anisomerous MI states using 16 EEG sensors. First, we combined the Butterworth band-pass filter, wavelet transform, and CCA to preprocess the raw EEG signals. We then used the R-CSP algorithm to extract feature vectors in the spatial domain. We subsequently utilized the KNN-SVM algorithm for classification. For comparison, five mainstream classifiers were used to classify the same sample data. The results indicate that the KNN-SVM classifier is more suitable for the recognition of the four MI states than the five mainstream classifiers. KNN-SVM also exhibits comparatively excellent results. In particular, the average classification accuracy rate is 87%, and the maximum accuracy rate is 92.5%. Based on these findings, we will assign the subjects to receive systematic MI training in the next stage. Thus, the proposed MI pattern recognition system reaches its maximum performance and satisfies the actual needs.

## Figures and Tables

**Table 1:** Classification results using CSP and R-CSP.

Subject	A	B	C	D	E
CSP AC (%)	80	85	85	82.5	85
Training set	200	200	200	200	200
Test set	40	40	40	40	40

R-CSP AC (%)	85	92.5	80	92.5	85
*β*	0.9	0.2	0.2	0.3	0.1
*γ*	0.5	0.9	0.4	0	0.3
Training set	1000	1000	1000	1000	1000
Test set	40	40	40	40	40

**Table 2:** Classification results of KNN-SVM.

Subject	R	RF	LH	RS
A	100	80	60	100
B	90	90	90	100
C	90	90	70	70
D	100	100	70	100
E	80	100	80	80
AC (%)	92	92	74	90

**Table 3:** Classification results from different classifiers.

Classifier	LDA	Random forest	Naive Bayes	KNN	SVM	KNN-SVM
Subject A	77.5	80	72.5	80	82.5	85
Subject B	80	85	65	82.5	82.5	92.5
Subject C	72.5	60	55	67.5	65	80
Subject D	87.5	85	85	92.5	87.5	92.5
Subject E	75	72.5	70	72.5	70	85
Average AC (%)	78.5	76.5	69.5	79	77.5	87
Standard deviation	5.7554	10.5475	10.9545	9.6177	9.5197	5.4199

**Table 4:** Comparison between the proposed method and previous studies for MI recognition.

Author	Electrode number	State number	Analysis method	AC (%)
Brunner [17]	22	4	CSP, LDAs	65.1
Lu [33]	64	3	SCS-NMF, SVM	68.9
García-Laencina [34]	4	2	BP/HJ/AAR, LFDA	77.3
Yi [4]	64	7	CAR, CSP, SVM	70
This study	16	4	wCCA, R-CSP, KNN-SVM	87
